# Influence of the incremental step size in work rate on exercise response and gas exchange in patients with pulmonary hypertension

**DOI:** 10.1186/1471-2466-8-3

**Published:** 2008-02-23

**Authors:** Sven Gläser, Sven Lodziewski, Beate Koch, Christian F Opitz, Henry Völzke, Ralf Ewert

**Affiliations:** 1 Division of Cardiology and Pneumology, University of Greifswald, Greifswald, Germany; 2 Department of Cardiology, DRK-Kliniken Berlin|Westend, Berlin, Germany; 3 Institute of Epidemiology and Social Science, University of Greifswald, Greifswald, Germany

## Abstract

**Background:**

Cardiopulmonary exercise testing (CPET) has become increasingly important as a routine procedure in daily clinical work. So far, it is generally accepted that an individualized exercise protocol with exercise duration of 6 to 12 minutes is preferable to assess maximal exercise performance. The aim of this study was to compare an individualized NYHA adapted exercise protocol with a fixed standard protocol in patients with severe pulmonary arterial hypertension.

**Methods:**

Twenty-two patients (17 female, 5 male; mean age 49 ± 14 yrs) underwent symptom limited CPET on a bicycle. On two consecutive days each subject performed a stepwise CPET according to a modified Jones protocol (16 Watt per minute stages) as well as an individualized NYHA adapted protocol with 5 or 10 Watt/min stages in a randomized order. Oxygen uptake at peak exercise (peakVO_2_) and anaerobic threshold (VO_2_AT), maximal ventilation (VE), breathing reserve (VE/MVV), ventilatory efficiency (VE vs. VCO_2 _slope), exercise time, maximal power and work rate were assessed and compared between both protocols.

**Results:**

Comparing both, adapted NYHA protocol and standardized Jones protocol, we found significant differences in maximal power (56.7 ± 19 W vs. 74 ± 18 W; p < 0.001) and exercise time (332 ± 107 sec. vs. 248 ± 72 sec.; p < 0.001). In contrast, no significant differences were obvious comparing both protocols concerning work rate, VE, VE/MVV, peakVO_2_, VO_2_AT and VE vs. VCO_2 _slope.

**Conclusion:**

Variations of incremental step size during CPET significantly affect exercise time and maximal power, whereas relevant parameters for clinical judgement and prognosis such as oxygen uptake, ventilation and ventilatory efficiency remain unchanged. These findings have practical implications for the exercise evaluation of patients with pulmonary hypertension. To reach maximal results for ventilation, oxygen uptake and gas exchange an individualization of incremental step size appears not to be mandatory.

## Backround

Cardiopulmonary exercise testing (CPET) has become increasingly important as a routine procedure in daily clinical work. It has been applied to patients with chronic congestive heart failure for risk stratification [1] and to assess responses to therapeutic interventions [2-5]. Parameters such as oxygen uptake at anaerobic threshold (VO_2_AT) and at peak exercise (peakVO_2_) are powerful predictors for survival in patients with advanced congestive heart failure [6]. Recently, its importance has been widened to patients suffering from severe pulmonary hypertension and right heart failure [7]. In addition to oxygen uptake related parameters, the characterization of ventilatory efficiency, usually expressed as the relation of minute ventilation to carbon dioxide output (VE vs. VCO_2 _slope), has become increasingly important due to its prognostic and therapeutic implications [8,9].

So far, it is generally accepted that an individualized exercise protocol is preferable to assess maximal exercise performance. Wasserman and colleagues introduced ramp or 1-minute incremental exercise tests with an "optimal" exercise duration of 6 to 12 minutes [10]. Therefore, an individualization of the increments in work rate is supposed to be mandatory in CPET to achieve optimal results for parameters of peakVO_2 _as well as ventilatory parameters.

The aspired individualization of the exercise protocol however, complicates the use of CPET in daily routine. The postulation of exercise durations between 6 and 12 minutes is mainly based on studies that investigated small groups of healthy volunteers [11-13]. There are concerns about the transfer of these results to patients suffering from pulmonary or cardiac diseases. Myers et al. found differences in parameters of peakVO_2 _in healthy subjects with different exercise protocols. However, they could not show this dependence in a small group of patients with congestive heart failure (CHF) [14]. In addition, the need of protocol individualization has to be debated in view of the data by Debigare et al. [15]. In this study the results of CPET were independent of different protocol designs in a group of 10 patients suffering on chronic obstructive pulmonary disease (COPD).

The aim of this study was to compare an individualized NYHA adapted exercise protocol with a fixed standard protocol in patients with severe pulmonary hypertension and right heart failure using a randomized protocol. The investigators hypothesized that there are no differences between the individualized to the "one-protocol-fits all" approach.

## Methods

### Study subjects

Seventeen female (mean age 46.9 ± 12.7 yrs) and five male (mean age 57.47 ± 15.1 yrs) subjects volunteered to participate in the investigation. All patients suffering from pulmonary arterial hypertension (WHO Group I [16]). Only patients with primary pulmonary hypertension were included. The mean systolic pulmonary artery pressure (PAP), estimated by echocardiography, was 68 mmHg ± 35 mmHg. No patients had echocardiographical evidence for a relevant left heart failure or underlying valvular disease. All patients were clinically assessed as NYHA/WHO class II (n = 10) or III (n = 12). They had to be clinically stable for at least 4 weeks prior to inclusion and continued their chronic medication. This targeted therapy included inhaled Iloprost (n = 5), Bosentan (n = 18), Sitaxsentan (n = 2), Sildenafil (n = 5), or Tadalafil (n = 2). In 11 patients combinations of these substances were given. None of the patients was on oxygen therapy at home.

The study protocol was approved by the Ethics Committee of the University of Greifswald. All subjects gave informed written comment.

### Exercise testing

Each subject performed two exercise tests in randomized order on two consecutive days. Consecutive patients were randomized to either protocol sequence in a one-to-one order. All subjects were requested to abstain from food, coffee and cigarette smoking for at least 3 hours prior to the test. All patients were assessed during there first clinical evaluation and were naïve to CPET.

The following bicycle tests were employed:

1. a symptom limited exercise test according to the Jones protocol [17] (stepwise increase in work load of 16 Watt/minute, starting with unloaded cycling plus the ergometer related permanent load)

2. a symptom limited exercise test according to an adapted NYHA protocol [10,18,19].

a. Protocol NYHA II: ramp test with an increase in work load of 10 Watt per minute, starting with unloaded cycling plus the ergometer related permanent load.

b. Protocol NYHA III: ramp test with an increase in work load of 5 Watt per minute, starting with unloaded cycling plus the ergometer related permanent load.

The assignment to either NYHA II or III protocol occurred according to clinical classification.

Each protocol was preceded by a resting period of at least 3 minutes to reach steady state conditions (steady state status was analyzed for VO_2_, VCO_2_, VE, EQO_2_/CO_2_, PETO_2_/CO_2_).

In the absence of chest pain, ECG abnormalities, complex arrhythmias or critical blood pressure changes all tests were continued as symptom limited (volitional exertion, dyspnoea or fatigue). Prior to the test patients were encouraged to reach maximal exhaustion, while during exercise no further motivational interventions were obtained. The protocol type was employed in a blinded fashion to the patient. After the test patients were asked to state the reasons for termination.

All tests were applied according to current guidelines for exercise testing [20] with continuous monitoring of ECG, blood pressure and oxygen saturation. All tests were performed at room air.

### Gas exchange variables

Respiratory gas exchange variables were measured continuously throughout the resting period, the unloaded cycling period and the exercise test using a VIASYS HEALTHCARE system (Oxycon Pro, Rudolph's mask. Prior to each test, the equipment was calibrated in standard fashion with reference gas and volume calibration. Standard 12-lead ECGs were obtained at rest, every minute during exercise, and for at least 5 minutes during recovery; blood pressure was measured with a standard cuff sphygmomanometer. Minute ventilation (VE), tidal volume (Vt), oxygen uptake (VO_2_) and carbon dioxide output (VCO_2_) were acquired on a breath-by-breath basis and averaged over 10 second intervals. Peak oxygen uptake (peak VO_2_) was defined as the highest 10-s average of oxygen uptake in the last minute of exercise. Ventilatory efficiency, expressed as the relation of VE and VCO_2_, has been assessed as the slope of the regression of both parameters excluding excess hyperventilation at the end of exercise. Previous work by our group and others has shown that this method of calculating the VE vs. VCO_2 _slope is prognostically optimal [1,3,8,21,22]. The anaerobic threshold was determined by two independent experienced observers in a blinded fashion according to Wasserman et al. [10]. The breathing reserve (VE/MVV) was calculated as maximal ventilation (VE) in relation to maximal voluntary ventilation (MVV). MVV was calculated by FEV1 × 41.

On maximal exercise we assessed the maximal power in Watt as shown by the bicycle ergometer; work rate was assessed by the product of exercise time and maximal power in Joule (J).

### Statistical analysis

All statistical analyses were performed with SPSS software, version 14.0.1 (SPSS GmbH Software, Munich, Germany). The means ± standard deviations (SD) and the confidence intervals of the means were calculated for peakVO_2_, VO_2_AT, VE, VE/MVV, maximal power, work rate, exercise time and VE vs. VCO_2 _slope. Paired sample t test was used to determine whether there was a significant difference between the means generated under two different exercise protocols. P values ≤ 0.05 were defined as statistically significant.

## Results

**Figure 1:**
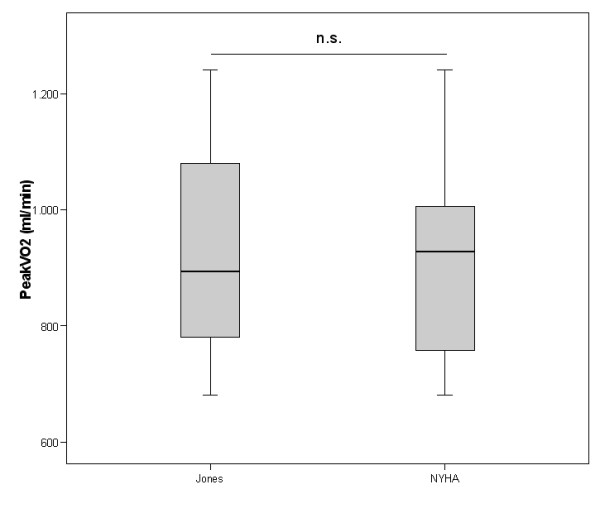
PeakVO_2 _(ml/min) assessed by NYHAII/III protocol and the Jones protocol indicated as mean, interquartile range and 97.5 confidence interval. n.s. for missing significance. 95% confidence interval for the difference of variables was -84.7 – 98.8.

**Figure 2:**
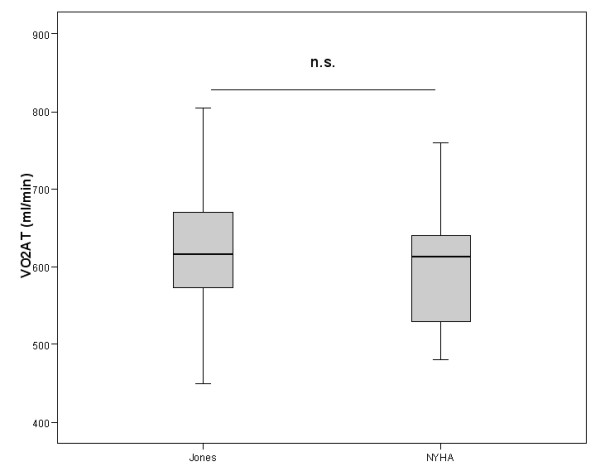
VO_2_AT(ml/min) assessed by NYHAII/III protocol and the Jones protocol indicated as mean, interquartile range and 97.5 confidence interval. n.s. for missing significance. 95% confidence interval for the difference of variables was -37.2 – 70.3.

**Figure 3:**
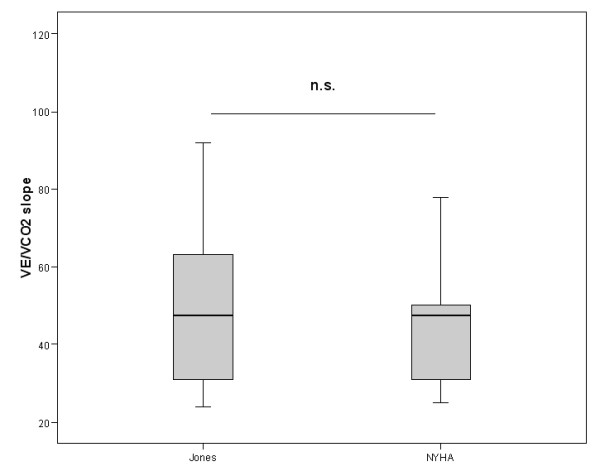
VE vs. VCO_2 _slope assessed by NYHAII/III protocol and the Jones protocol indicated as mean, interquartile range and 97.5 confidence interval. n.s. for missing significance. 95% confidence interval for the difference of variables was -4.6 – 7.4.

In the study population 10 patients were assigned to the protocol NYHA II, 12 to the protocol NYHA III. According to the randomization sequence an equal split for either starting with the NYHA or Jones protocol was reached. There were no significant differences comparing day 1 and day 2 CPET regarding peakVO_2 _(938 ± 190 vs. 934 ± 192 ml/min) and VO_2_AT (633 ± 108 vs. 620 ± 108 ml/min). In two patients assigned to NYHA III protocol the anaerobic threshold could not be determined neither by the NYHA nor by the Jones protocol procedure according to the criteria mentioned above. In all other cases the calculation of the anaerobic threshold was supported by at least two corresponding methods. Significant differences between NYHA and Jones protocol procedures were obvious for exercise time and maximal power. No significant differences were found for parameters describing exercise performance (peakVO_2 _and VO_2_AT), ventilation on exercise (maximal VE, VE/MVV) as well as ventilatory efficiency (VE vs. VCO_2 _slope) (see Table 1, 2 and Figures 1, 2, 3). Comparing the CPET according to the Jones protocol to the corresponding NYHA II and III subgroup again no significant differences were obvious for peakVO_2_, VO_2_AT, VE vs. VCO_2 _slope, maximal VE and VE/MVV.

**Table 1:** Comparison of Jones and NYHA CPET protocol for the entire groups^*^

	Protocol	Mean ± SD	
Resting blood pressure [mmHg] systolic/diastolic	NYHA (II/III)	111 ± 28.3/80.5 ± 6.4	p < 0.001
	Jones	120.5 ± 24/85.5 ± 12	
Peak blood pressure [mmHg] systolic/diastolic	NYHA (II/III)	120 ± 27/73 ± 21	n.s.
	Jones	130.4 ± 21/78 ± 17	
Heart rate at rest [1/min]	NYHA (II/III)	87.1 ± 11.7	
	Jones	88.7 ± 10.4	n.s.
Peak heart rate [1/min]	NYHA (II/III)	128.2 ± 22.6	
	Jones	128.7 ± 19.2	n.s.
SaO_2 _[%] at rest	NYHA (II/III)	95 ± 6	
	Jones	96 ± 5.5	n.s.
Peak SaO2 [%]	NYHA (II/III)	88 ± 6.4	
	Jones	86 ± 6.9	n.s.

^*^Mean and standard deviation [SD]. n.s. for missing significance. SaO_2 _for arterial oxygen saturation measured by oxymetry.

**Table 2:** Comparison of Jones and NYHA CPET protocol for the entire groups^*^

	Protocol	Mean ± SD	[95% CI's]
Maximal power [W]	NYHA (II/III)	56.77 ± 18.98	[10.9–24.6]
	Jones	74.55 ± 18.25	p < 0.001
Work rate [J]	NYHA (II/III)	14566.48 ± 8241.8	[-5450.4 – 639.3]
	Jones	12160.9 ± 5694.8	n.s.
Exercise time [sec.]	NYHA (II/III)	332 ± 107	p < 0.001
	Jones	248 ± 73	
VE [l]	NYHA (II/III)	48.5 ± 14.9	[-2.7 – 4.1]
	Jones	49.23 ± 16.1	n.s.
VE/MVV	NYHA (II/III)	0.567 ± 0.21	[-0.03 – 0.06]
	Jones	0.58 ± 0.23	n.s.

^*^Mean and standard deviation [SD] and 95% confidence intervals [CI's] for the differences between both groups. n.s. for missing significance.

Reasons for terminating exercise were dyspnoea in 16 patients (including all patients assessed as NYHA class III) and dyspnoea in addition to general fatigue in 6 patients. The reason for terminating exercise did not differ between day 1 and 2 in any patient. All tests were performed symptom limited, no premature termination by the investigator occurred.

## Discussion

Aim of the study was to compare different exercise protocols in patients suffering from significant cardiac limitations due to pulmonary hypertension. Currently, the need of an individualized exercise protocol to reach exercise durations between 6 and 12 minutes is generally accepted. In daily practice, protocols according to the recommendations of Wasserman et al. [10] are widely used to reach exercise durations between 6 and 12 minutes. These recommendations are mainly based on data obtained in healthy volunteers [11-13], however, the necessary calculations providing individually adjusted exercise protocols complicate the daily routine of this method.

Data by Myers et al. collected within a small group of CHF patients comparing different exercise protocols (cycle ergometry), show ignorable differences for prognostically and clinically relevant parameters such as peakVO_2 _and VO_2_AT [14]. Debigare et al. showed comparable results in a small group of COPD patients.

Similarly, our data did not show a major impact of the exercise protocol design on peakVO_2 _and VO_2_AT in patients suffering from pulmonary hypertension. Comparing the results for day 1 and day 2 exercise tests significant differences concerning oxygen uptake and therefore exercise tolerance were not seen. Thus, we assume an ignorable influence of the previous test onto the following procedure. In addition, ventilatory efficiency – as expressed as VE vs. VCO_2 _slope – was not affected by different exercise protocols. Therefore, the majority of clinical and prognostic parameters with relevance to patients with severe cardiac limitations were independent of different exercise protocol designs.

Changes in exercise duration and maximal power output are related to the size of the incremental steps used in various exercise protocols.

An increasing step size is physiologically accompanied by shorter exercise durations and higher maximal work loads. Maximal work load, exercise duration and power however, are not related to prognosis in patients with pulmonary hypertension in a multivariate analysis [23]. Comparing both protocols (NYHA and Jones) we did not find differences in the quality of anaerobic threshold determination using the methods introduced by Wasserman et al. Even though this study was not designed to evaluate this topic we completely agree with the need of one minute incremental steps or continuous progression of protocols with respect to the quality of anaerobic threshold determination. The exercise duration as well as the exercise protocol did not influence the reproducibility of the anaerobic threshold determination.

Corresponding to the results of oxygen uptake und ventilatory efficiency in relation to the exercise protocol used ventilatory parameters such as maximal ventilation and breathing reserve remained unaffected by the choice of protocol. Although our study was not conducted to evaluate patients with significant ventilatory limitations these observations correspond to the results published by others [11,12,14,15].

Our study has several limitations. First, we decided to compare a limited number of protocols in a well defined group of patients. Whether these results can be applied to other protocol designs and to patients with other than cardiac limitations remains to be shown. Second, the exercise time in the NYHA adapted tests did not reach the currently recommended lower limit of 6 minutes in all cases. Therefore, the expected exercise capacity had been overestimated by the NYHA adaptation in a number of patients. We assume however, that this problem is also common in daily clinical work. Finally, the number of patients limits the study in its power. By obvious technical reasons as well as in agreement with the approval by the ethics committee the study was undergone in a single blinded fashion resulting in a blinding to the patient. However, the resulting bias was minimized by the commitment to encourage the patients only prior to exercise in always the same way.

## Conclusion

To our knowledge, these are the first data prospectively comparing two different exercise protocols in a relevant number of patients with severe cardiac limitation due to pulmonary hypertension. Focusing on the clinically and prognostically relevant parameters of oxygen uptake at anaerobic threshold and peak exercise as well as on ventilatory efficiency we did not find relevant differences. In conclusion, using a uniform exercise protocol with one minute incremental steps based on Jones et al. appears to be sufficient in these patients. Concerning these parameters an individual protocol adaptation is not superior to a simplified approach.

## Competing interests

All contributing authors state to have no competing financial or non-financial interests.

## Authors' contributions

SG – wrote the manuscript, coordinated and designed the study and approved the final version

SL – was involved in drafting the manuscript, proceeding the graphs and helped in the acquisition of the data and statistical analysis

BK – carried out data acquisition and was involved statistical analyses

CFO – was involved in study design and drafting the manuscript

HV – was involved in study design and drafting the manuscript

RE – was involved in study design and drafting the manuscript

All authors approved and read the final version of the manuscript.

## Pre-publication history

The pre-publication history for this paper can be accessed here:


